# A new detector for sub-millisecond EXAFS spectroscopy at the European Synchrotron Radiation Facility

**DOI:** 10.1107/S1600577514014805

**Published:** 2014-10-03

**Authors:** Innokenty Kantor, Jean-Claude Labiche, Emmanuel Collet, Laurent Siron, Jean-Jacques Thevenin, Cyril Ponchut, Jacques Borrel, Trevor Mairs, Carlo Marini, Cornelius Strohm, Olivier Mathon, Sakura Pascarelli

**Affiliations:** a European Synchrotron Radiation Facility, 71 avenue des Martyrs, 38043 Grenoble, France; b CELLS-ALBA, Carretera, BP 1413, Cerdanyola del Vallès 08390, Spain

**Keywords:** pixel array detectors, charge-coupled devices, EXAFS

## Abstract

A new FReLoN (Fast-Readout Low-Noise) high-frame-rate detector adopted for the fast continuous collection of X-ray absorption spectra is presented. The detector is installed on the energy-dispersive X-ray absorption beamline ID24 at the ESRF and is capable of full time-resolved EXAFS spectra collection with over 4 kHz repetition rate and 0.2 ms exposure time. An example of the *in situ* kinetic study of the high-temperature oxidation of metallic iron is presented.

## Abstract 2

The design and performance of the new sub-millisecond detector for time-resolved X-ray absorption spectroscopy at ID24 at the ESRF is described.

## 1. Introduction   

The development of third-generation synchrotrons, with their insertion devices and X-ray optics, is constantly improving the characteristics of X-ray beams available for research in terms of photon flux, emittance, coherence and spectral properties. Various types of time-resolved studies are becoming more and more common in modern synchrotron-based science. They can be divided into those using several repetitions of reproducible events and those recording a single event. As an example of the first type of experiment one can mention X-ray diffraction in a pulsed laser-heated diamond-anvil cell (Goncharov *et al.*, 2010 ▸). For this type of experiment, the detector does not necessarily need a high repetition rate; however, the exposure time is required to be short enough to obtain a high time resolution. Furthermore, there is no limitation on the signal intensity, since the required statistics can be obtained by summing multiple repeating measurements. In many cases, a high repetition rate is also important: for example, for experiments in pulsed magnetic fields (Strohm *et al.*, 2012 ▸), where measurements at different times during the field pulse are desired. An example of the second type of experiment is shock compression study of matter, where the sample is destroyed after a single shock event (Gupta *et al.*, 2012 ▸). For this type of measurement, to follow the changes in the sample with time, both a high repetition rate and a short exposure time of the detector are required. In addition, the detector sensitivity and the photon flux should be high enough to provide sufficient statistics for each individual spectrum or measurement in a sequence.

In many situations, the limitations in time-resolved studies are no longer imposed by the photon flux, but rather through the detector (which can be limited by its sensitivity, readout or data-transfer time). The recently upgraded X-ray absorption beamline ID24 at the ESRF is dedicated to fast time-resolved and extreme-conditions X-rays absorption spectroscopic (XAS) studies and is demanding for a fast, low-noise and high-dynamic-range X-ray position-sensitive detector. X-ray absorption spectroscopy is a powerful tool for studying electronic and magnetic properties, local structure and valence state of a specific element in ordered (crystalline) as well as disordered (amorphous or liquid) and heterogeneous systems. The energy-dispersive spectrometer on ID24 has a huge advantage for fast spectral collection, since the whole spectrum is collected at once, without the need for scanning or moving any X-ray optical element, providing very high spatial and temporal beam stability (Pascarelli *et al.*, 2006 ▸).

For the needs of ID24 and other ESRF beamlines, a special fast-readout low-noise (FReLoN) detector has been developed. The first incarnation of the FReLoN camera for fast time-resolved studies was presented more than five years ago (Labiche *et al.*, 2007 ▸), and was based on a two-dimensional 2048 × 2048 pixels CCD chip. Here we present a new, improved, version of the FReLoN detector based on a linear CCD array that has much faster readout and higher repetition rate, and also can accept a wider X-ray beam, allowing full exploitation of the improved capabilities of the new ID24 beamline, as described below.

## 2. Geometrical constraints for detection using an energy-dispersive spectrometer   

**Figure 1:**
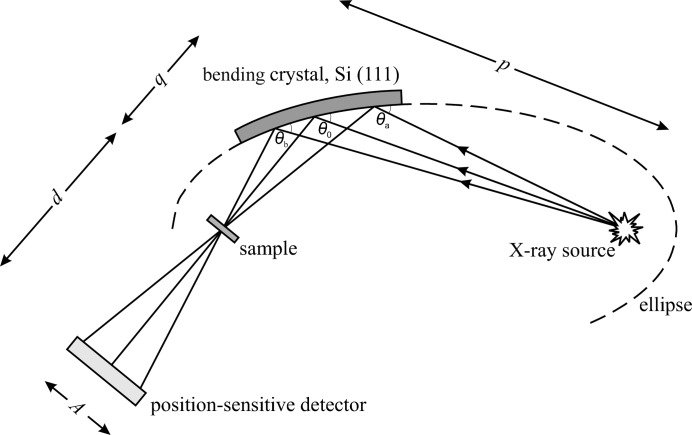
The optical scheme of the energy-dispersive XAS spectrometer. On beamline ID24 the source is a demagnified image of the undulator source (*i.e.* secondary source). Source-to-crystal distance (*p*) is equal to 22 or 31 m (depending on the chosen beamline branch), *q* = 0.6–1.2 m and *d* = 1.2–2.4 m.

In the energy-dispersive scheme used at ID24, as illustrated in Fig. 1 ▸, a curved crystal focuses a polychromatic fan of X-ray radiation onto the sample position, introducing at the same time a correlation between the photon energy and the direction of propagation; *p* and *q* are, respectively, the source–crystal and crystal–focal-spot distances. The angle covered by the polychromatic fan impinging on the sample is Δψ_fan_ = *L*/*q*, where *L* is the horizontal dimension of the beam intercepted by the polychromator (*L* ≃ 50 mm on ID24). The full spectral range diffracted by the crystal, Δ*E*, is proportional to the variation of Bragg angle θ along the beam footprint on the crystal, Δθ:
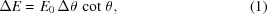
where *E*_0_ is the central energy. On ID24, the maximum value of the ratio Δ*E*/*E*_0_ ranges from 8% at the lowest energy (5 keV) to 20% at high energies (>12 keV).

The transmitted beam is detected on a position-sensitive detector where energy is correlated to position. The angular acceptance of the detector, Δψ_acc_ = *A*/*d*, where *A* is the horizontal aperture and *d* the detector–focal-plane distance, determines the portion of the total energy range detected, Δ*E*_detected_/Δ*E*: 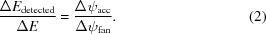
It is particularly important to detect the full spectral range (Δ*E*_detected_/Δ*E* = 1) at low energies, where the energy bandwidth of the polychromator is less than 10%.

On the upgraded version of ID24, efforts were made to reduce *q* as much as possible, in order to increase Δ*E* [increasing Δθ, from equation (1)], leading to a maximum Δψ_fan_ = 70 mrad. This implies that, in order for the detector to intercept the full diffracted polychromatic fan, Δψ_acc_ needs to also reach a maximum of 70 mrad.

The point spread function Δ*r* (a function of horizontal pixel size) as well as the distance of the detector from the focal point *d* determines δψ_detector_, the angular acceptance of each pixel:
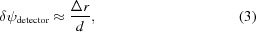
where δΨ_detector_ is proportional to the detector contribution to the energy resolution of the measured spectrum, δ*E*_detector_. Equations (2) and (3) show that in order to cover the required energy range Δ*E*_detected_ without compromising energy resolution, it is important to have a detector with a large horizontal aperture *A*. The criteria for the choice of *A* and *d* were based on maintaining δ*E*_detector_ smaller than other contributions to the total energy resolution: the intrinsic energy resolution of the polychromator (*i.e.* determined by the Darwin width) and the contribution from the finite source size. At low energies, the dominant contribution to energy resolution derives from the intrinsic energy resolution of the chosen polychromator. For example, when working at the Fe *K*-edge (7.1 keV), the energy resolution given by the intrinsic polychromator resolution is about 0.7 eV, while the energy resolution defined by the pixel size is about 0.2–0.3 eV. The δ*E*_detector_ contribution to the energy resolution is highest at energies around 12–13 keV; however, it is always smaller than the intrinsic polychromator resolution.

Because of the horizontal X-ray beam dispersion, the spectral energy range Δ*E*, the contribution δ*E*_detector_ to the energy resolution and the number of photons per pixel can be adjusted by varying *d*, *i.e.* moving the whole detector along the beam propagation direction, closer or further away from the focal spot.

The intensity distribution along different energies in a spectrum is not uniform. It is defined by the emission spectra of the undulators, and by the throughput of all X-ray optics (mirrors and polychromator) and windows of the beamline. By changing the undulator gaps one can adjust the intensity distribution along the spectrum. Typically intensity varies by 30–40% over the whole energy range.

## 3. Optical design of the new FReLoN   

**Figure 2:**
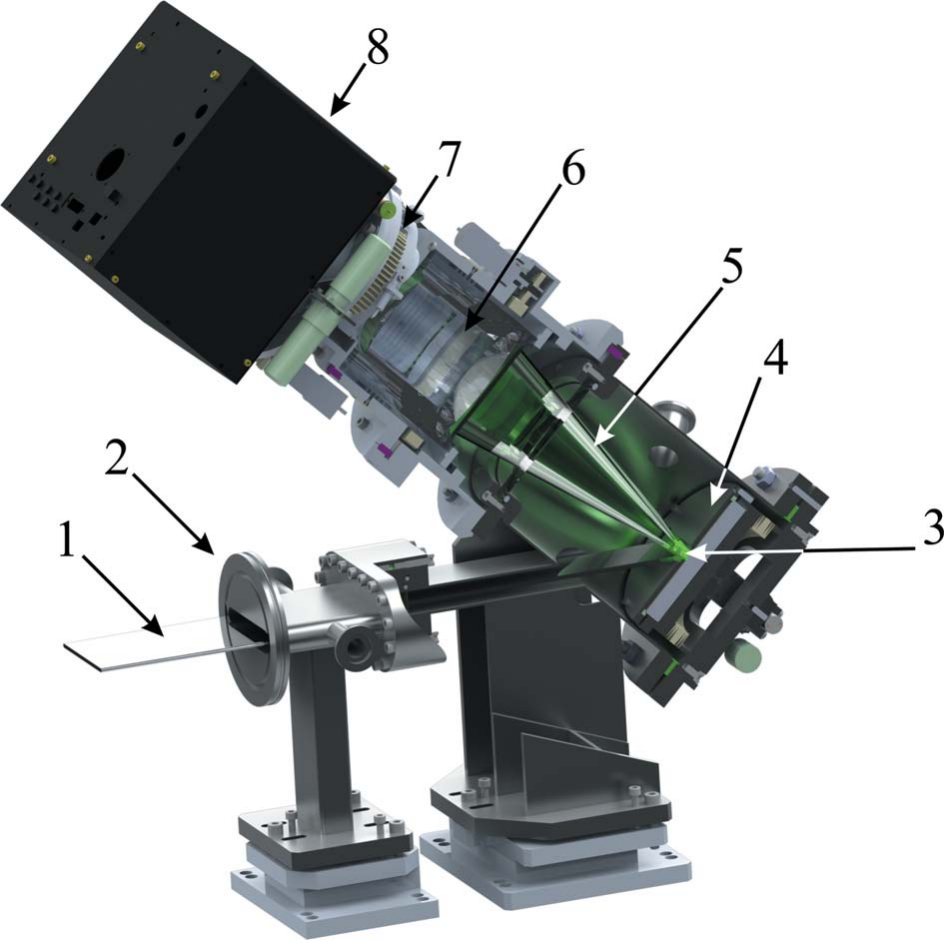
The optical scheme of the new position-sensitive detector (partially cut view): 1 is the flat horizontally diverging X-ray beam, 2 is the vacuum tube connection flange, 3 is the fluorescence screen for the conversion of X-rays to visible photons, 4 is the metallic slot mask for the fluorescence screen, 5 is the visual light cone accepted with the wide-angle optics (6), 7 is the motorized iris aperture assembly, 8 is the FReLoN camera housing.

The X-ray photons are detected indirectly: first, the X-rays are absorbed on a scintillator screen and converted to visible light (Fig. 2 ▸). The visible light is then detected by a charge-coupled linear array device. This scheme presents several advantages: the scintillator screen material can be chosen depending on the specific X-ray energy range and the required time resolution, as different scintillator materials have different sensitivities and after-glow characteristics. The screen can be easily replaced if deteriorated by the X-ray beam, the silicon CCD camera operates at its maximum quantum efficiency (visible photons range), and the camera chip is protected from possible damage through direct X-ray beam exposure.

The optical scheme of the FReLoN detector is shown in Fig. 2 ▸. This new version of the detector with newly designed optics can accept a fan of X-rays (1) with a horizontal width up to 100 mm. X-rays enter the detector from a vacuum beam flight-tube attached to the entrance flange (2). The scintillator screen (3) is mounted at 45° with respect to the plane of the incident X-ray fan. A metallic mask (4) with a slot placed just in front of the scintillator screen prevents scattered X-rays from exciting unwanted fluorescence. Visible light (5) is then collected and projected on the CCD camera through a demagnifying tandem lens system (6) consisting of a large field custom objective on the scintillator side and a wide aperture Zeiss Planar 1.4/85 ZF objective on the camera side, providing an ultra-sharp and undistorted image over the entire field of view. A motorized iris aperture (7) allows one to adjust the amount of light transmitted to the camera. Since each X-ray photon can excite several visible photons, it is sometimes important to reduce the number of visible photons to achieve total quantum efficiency of the system close to unity, to prevent oversaturation and to control precisely the exposure time. The FReLoN camera (8) is connected on the top with easy access to all the interface cable connectors and water-cooling tubes for the Peltier element and the thermal stabilization of the electronic boards inside (see Labiche *et al.*, 2007 ▸, for more details).

For precise remote detector alignment on the beamline, two additional motorized movements (using stepper motors) are implemented in the detector: a tilt of the CCD camera around its optical axis for exact matching of the camera orientation to the image on the screen, and a motorized focus of the FReLoN objective. The modular design makes it very simple to change either the scintillator screen or the camera if necessary within a few minutes.

## 4. Linear CCD FReLoN camera: a multi-kilohertz linear detector   

The basic FReLoN platform is made of a camera head and a data acquisition board, both linked by a serial line fiber optic cable and a power supply unit.

**Figure 3:**
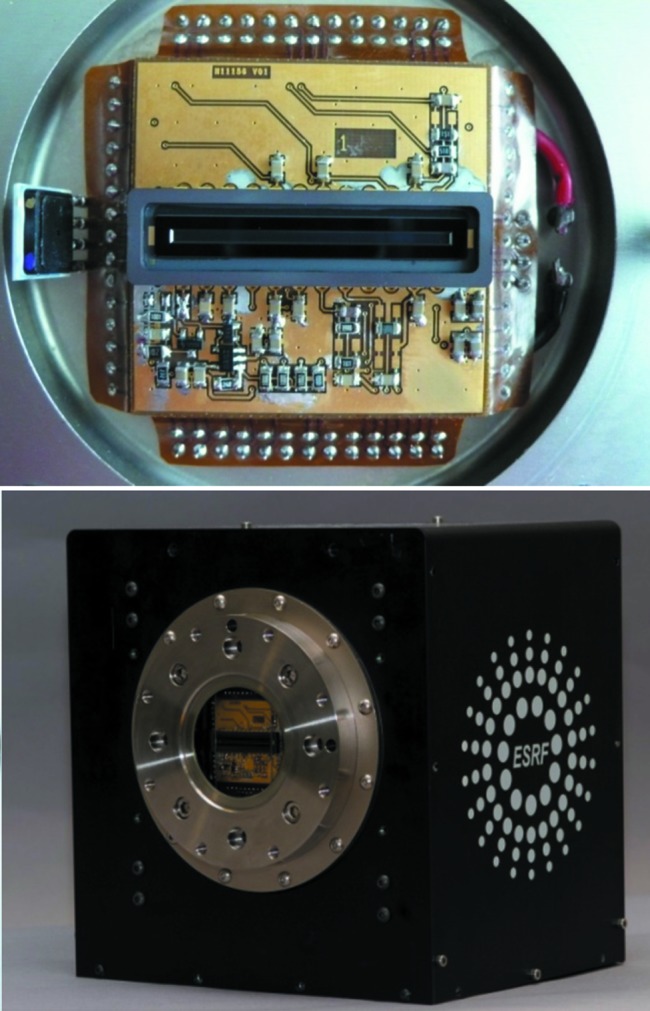
The S11156 Hamamatsu sensor integrated in the new FReLoN camera.

To fit with the objectives above, a new linear CCD image sensor 11156 from Hamamatsu, consisting of an array of 2048 pixels of 14 µm × 1000 µm, has been integrated in the well known FReLoN camera platform previously developed at ESRF (Fig. 3 ▸). A signal processing technique has been implemented based on the digital correlated double sampling (DCDS) method (see below).

The CCD is integrated into a vacuum chamber containing the thermoelectric cooler which maintains the CCD temperature at 256 K. The dark current is then reduced to a few electrons per pixel per second to allow exposures of microseconds to a few seconds without significant excess noise. Extreme care has been taken to ensure the shortest possible wires between the CCD chip and the clock drivers and signal preamplifiers. This was a prerequisite to cope with both fast rise time, high current clocks and low-level wide bandwidth signals from the CCD output.

The S11156-2048 chip utilizes a resistive gate structure that allows high-speed transfer with the ‘on chip’ electronic shutter function, offering significantly reduced image lag (less than 0.1%), even if the pixel height is large. With their back-thinned structure, these CCDs also offer a high sensitivity (>80% quantum efficiency) from the UV to the near-IR region of the spectrum.

The FReLoN camera is always ready for a new exposure without any dead time before starting a new integration. The jitter time of the synchronization is ±12 ns and the accuracy of the integration width is ±0.5 ns. This low level of jitter time (in ns) compared with the short exposure time (in µs) allows accurate acquisition time windows even in single-shot data acquisition.

**Figure 4:**
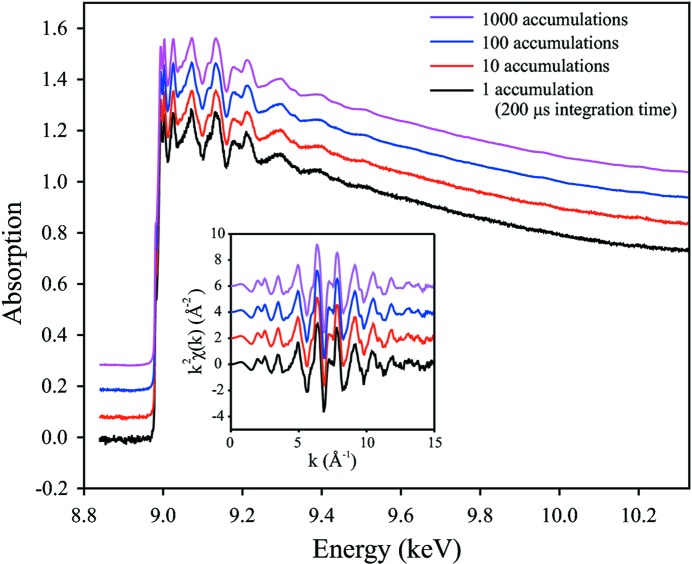
Quality of data collected on a pure Cu foil on ID24. The curves from bottom to top represent EXAFS spectra obtained from one, ten, 100 and 1000 accumulations of 200 µs each. The inset shows the extracted *k* ^2^-weighted EXAFS oscillations. All EXAFS features, even at the highest *k*-values, are already clearly visible on the single shot spectrum.

The plot in Fig. 4 ▸ shows Cu *K*-edge EXAFS spectra recorded on ID24 by averaging an increasing number of accumulations of 200 µs exposure time each. The noise in these spectra is dominated not by the counting statistics but rather by the fact that *I*_0_ (intensity distribution in the upcoming beam) and *I*_1_ (intensity distribution after the sample) are not recorded simultaneously, while the X-ray beam structure changes slightly with a high frequency. These intensity variations can be averaged out by taking more spectral accumulations. In the uniform filling of the storage ring, photon flux exceeds 10^6^ photons per pixel in a single exposure time of 200 µs.

### 4.1. Characterization and results   

#### 4.1.1. Data signal processing   

**Figure 5:**
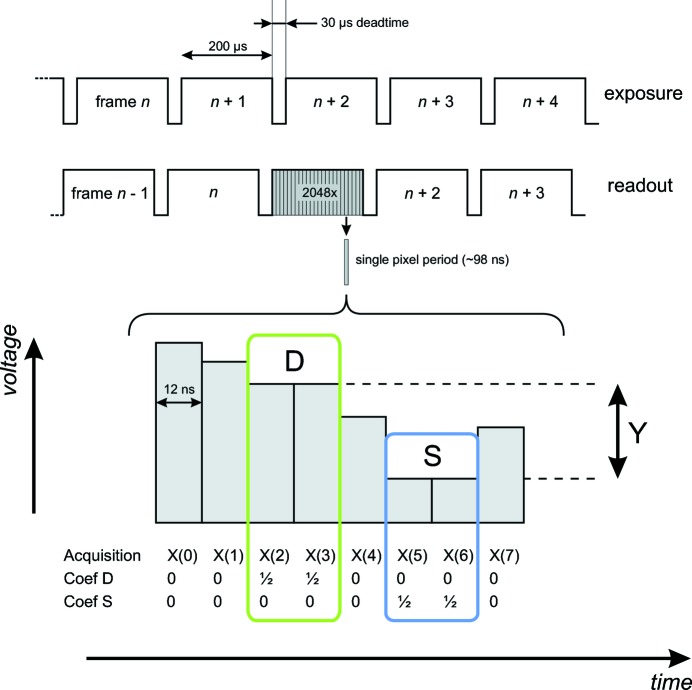
The upper panel shows the timing scheme of the camera exposure and readout. The lower panel shows the timing scheme of a single pixel period, explaining the principle of the DCDS: the video signal of the pixel period above is sampled *via* a fast ADC (analog-to-digital converter) and each sample [*X*(*i*)] is weighted with a coefficient (see text for explanation).

The timing scheme of the CCD camera at the highest frame rate is shown in Fig. 5 ▸ (top panel). With a dead-time of 30 µs and an exposure time of 200 µs, the camera is capable of continuous acquisition at 4.3 kHz. The data signal processing is done through DCDS. This technique proves to be a versatile method to optimize the performances of each sensor. The charge stored inside each pixel is converted by an on-chip charge preamplifier giving a floating voltage output that is digitized by a fast and accurate analog-to-digital converter and is sent to a field-programmable gate array. As a result, eight different sample readings are consequently measured for each pixel. Then each sample is associated with a weighted coefficient (Fig. 5 ▸, lower panel). The value of the charge is obtained by performing a digital difference between the reference signal (the D-zone floating diode) and the charge signal (S-zone signal). At the same time, an integral nonlinearity correction is added by using a look-up table.

The sum of *D* coefficients (the D-zone floating diode) is equal to unity, as well as the sum of *S* coefficients (S-zone charge signal). Final reading of the charge *Y* for each pixel is calculated as
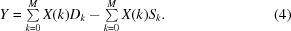
For the case shown in Fig. 5 ▸, the calculated value would be
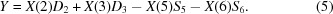



#### 4.1.2. Photon transfer curves   

To characterize this camera we have used the plot of the photon transfer curves (Janesick, 2001 ▸), which is the most explicit characterization of the CCD parameters. A photon transfer curve is obtained by taking a series of pairs of uniformly illuminated exposures, with varying number of photons for each pair. By analyzing the difference between each pair of images one can calculate the noise level σ and photonic variance σ^2^. By analyzing the photon transfer curve one can obtain all the characteristics of the camera such as readout noise, dark current generation, full-well capacity, linearity, sensitivity or dynamic range. More details on this technique can be found elsewhere (Janesick, 2001 ▸).

**Figure 6:**
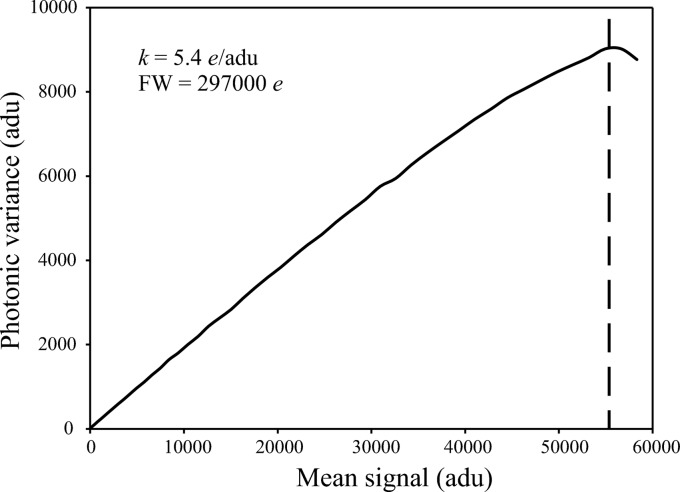
The photon transfer curve for the new FReLoN camera, showing the photonic variance (square of noise) as a function of mean camera output signal. The inverse of the slope is given by the camera gain *k*, and the saturation level defines the full-well capacity FW (see Table 1 ▸).

The plot of the variance (Fig. 6 ▸) gives the value of the full-well saturation (FW), the true dynamic range or the gain (*k*) in electrons per analog-to-digital unit (ADU) = mean signal/photonic variance. The values for FW and *k* are 297000 electrons and 5.4 electrons/ADU, respectively.

**Figure 7:**
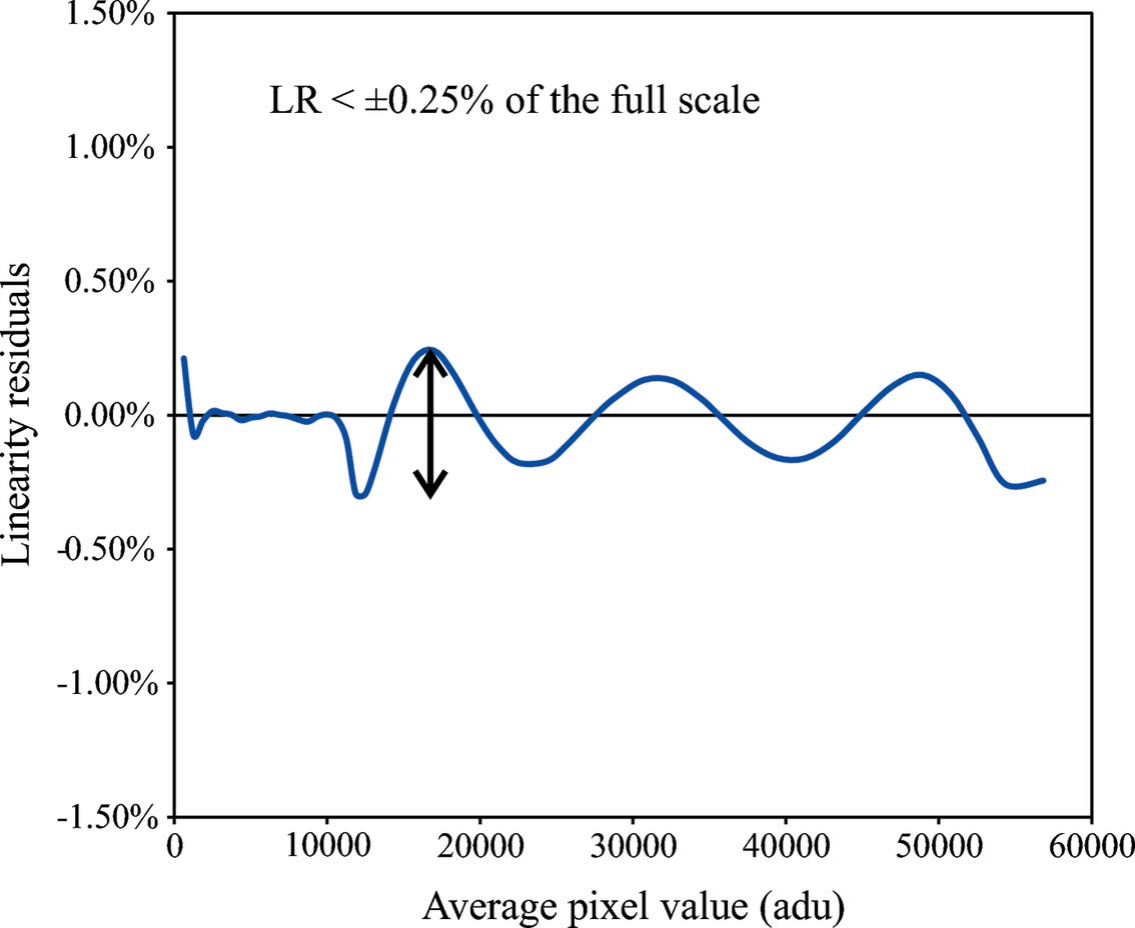
The linearity residuals of the new FReLoN linear CCD detector show a linearity better than ±0.25% of the full scale.

The CCD linearity is characterized by plotting the signal (ADU) as a function of the exposure time. When measuring nonlinearity below 1%, a specific plotting technique is used and defined as the linearity residuals (LR)
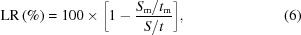
where *S*_m_ and *t*_m_ are the signal and time at the middle scale, and *S* is the signal at time *t*. The LR value does not exceed ±0.25% on the full scale (Fig. 7 ▸).

#### 4.1.3. Main performance characteristics of the FReLoN camera   

An overview of the main parameters of the new FReLoN camera is given in Table 1 ▸. The parameters are compared with those of the S11165 system, which is a driver circuit designed for this particular linear CCD array by the original manufacturer Hamamatsu. The R&D performed with the FReLoN system pushes the performance in two directions: the effective frame rate is improved by a factor of three and the true dynamic range by a factor of three. The electronic noise and dark current values are also significantly reduced.

## 5. Application example: *in situ* oxidation of iron above 1000 K   

As a demonstration of the ability of the new detector to perform full time-resolved kinetic studies in the sub-millisecond domain we have studied the oxidation of metallic iron in air at high temperature.

The chemical reaction Fe + O_2_ = Fe_2_O_3_ is responsible for iron corrosion at atmospheric conditions and is one that humans have dealt with since the Iron Age. Even the famous brown color of ancient Greek pottery appeared due to this reaction (Hofmann, 1962 ▸). Iron is a multi-valence metal and forms different oxides depending of the oxygen activity (fugacity) and temperature: wüstite Fe_*x*_O (non-stoichiometric oxide with predominantly Fe^2+^), magnetite Fe_3_O_4_ (with one third Fe^2+^ and two thirds Fe^3+^) and hematite Fe_2_O_3_ (all Fe^3+^). At normal atmospheric conditions [log(*f*O_2_) = −0.7] hematite is the only stable iron oxide phase (Ghiorso & Sack, 1991 ▸).

**Figure 8:**
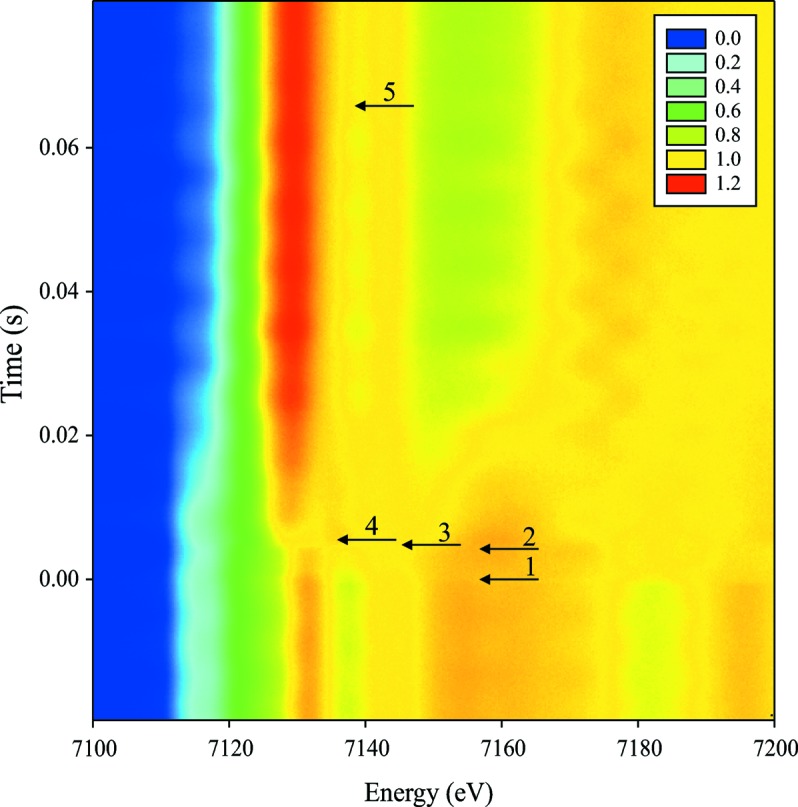
Normalized X-ray absorption near Fe *K*-edge as a function of time. Small black arrows indicate time of the following events: 1, heating laser turned on; 2, sample temperature reaches ∼1200 K and a phase transition from α-Fe to γ-Fe starts; 3, phase transition is finished, only a pure γ-Fe phase is observed; 4, first traces of iron oxide appear in spectra; 5, the oxidation is complete and the sample is fully converted to Fe_2_O_3_ hematite.

A pure Fe foil (99.85% from Goodfellow) of 5 µm thickness exposed to normal atmosphere was heated with a highly defocused (focal spot of ∼1 mm) 5 W IR laser hitting the foil at an angle of 25°. Heating was synchronized with detection *via* an analog TTL signal. The polychromatic X-ray beam was focused down to about 5 µm × 4 µm on the sample in order to minimize probed thermal gradients. Fig. 8 ▸ shows time and energy dependence of normalized X-ray absorption around the *K*-edge of iron (7.112 keV), recorded every 230 µs as a color map. Several time-stamps are drawn on the map as small horizontal arrows (see figure caption). Some periodic horizontal bands can be seen in Fig. 8 ▸ with a frequency of ∼120 Hz. These are related to the X-ray beam intensity variations in time during the measurements and most likely related to the electronic beam instabilities in the storage ring.

More than 400 spectra were collected in 0.1 s. They were then normalized and analyzed with a standard linear combination fit technique in order to extract the relative fractions of the principal components. The pure components spectra were extracted from the experimental sequence.

**Figure 9:**
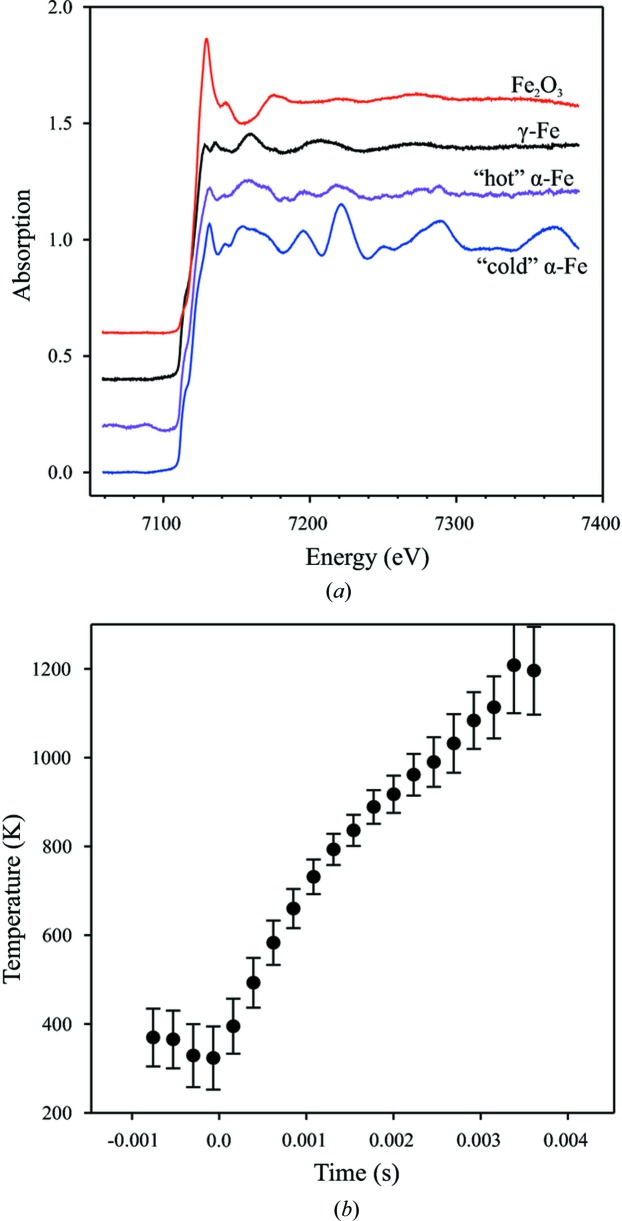
(*a*) XAS spectra of pure components used in the linear decomposition fit (shifted vertically for clarity). From bottom to top: blue, ‘cold b.c.c.’ (at room temperature) α-Fe corresponding to the time-stamp 1 in Fig. 8 ▸; pink, ‘hot b.c.c.’ (∼1200 K) α-Fe (time-stamp 2 in Fig. 8 ▸); black, γ-Fe (time-stamp 3 in Fig. 8 ▸); red, Fe_2_O_3_ hematite (time-stamp 5 in Fig. 8 ▸). (*b*) Temperature evolution of the α-Fe phase estimated from EXAFS fits. Zero on the timescale indicates the moment at which the laser is switched on.

During the heating of the α phase (with the body-centered cubic ‘b.c.c.’ structure) of iron, significant thermal damping of the EXAFS occurs. Therefore, we include two different components for the α-Fe: a cold b.c.c. and a hot b.c.c. phase. The ‘hot b.c.c.’ was taken just before the onset of the phase transformation to the high-temperature γ-Fe phase (with a face-centered cubic ‘f.c.c.’ structure) (Fig. 9 ▸ *a*).

Temperature was not measured directly in this experiment; however, we were able to estimate the temperature of the sample from the data analysis using a simple model. Sixteen individual EXAFS spectra of α-Fe were acquired between the moment the laser was turned on and the onset of the transformation to the γ phase. We performed a standard EXAFS analysis using the *FEFFIT* code (Newville, 2001 ▸). In the b.c.c. structure the first two coordination shells (eight nearest neighbors at *R*1 and six next-nearest neighbors at *R*2) are closely overlapped and cannot be separated in the Fourier transform, so we treat them simultaneously, putting a geometrical constraint on the *R*1 and *R*2 distances [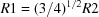], and using a Debye model for the thermal factors. With this model all spectra were fitted simultaneously, sharing a common effective Debye temperature (although this assumption is not totally true, it was good enough for the present case owing to the limited spectral *k* range and therefore large σ^2^ uncertainties). Individual temperatures were left as free parameters. The results of this fit are shown in Fig. 9 ▸(*b*). The highest temperature values obtained for the pure α phase are ∼1200 (100) K, perfectly matching within the uncertainty of the α → γ transition temperature of iron (1185 K). Therefore, we believe that this estimation is quite reasonable, and the observed trend seems to have a plateau at *T* ≃ 1300 K. We assume that the temperature remains relatively stable during the oxidation reaction.

**Figure 10:**
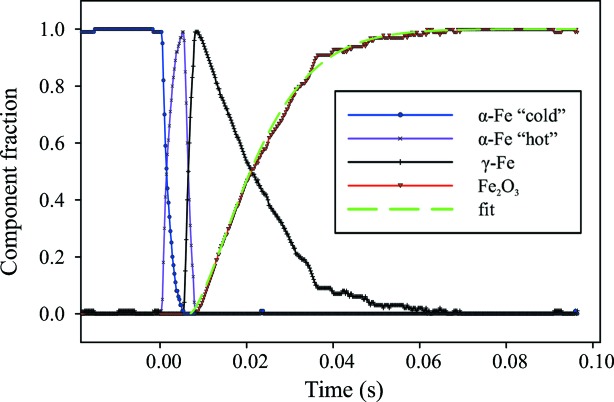
Relative fractions of the principal components as a function of time. The green dashed line shows a simple exponential fit; see text for details.

Fig. 10 ▸ shows the resulting relative fractions of the principal components representing the XAS spectra. The transformation of iron to iron oxide requires diffusion of oxygen inside the metal foil, and this reaction occurs significantly slower than the α- to γ-iron transition, that is limited only by nucleation and growth of the new phase and occurs within 2 ms. We would like to point out that we did not observe any metastable phases of iron oxides other than hematite in our experiment.

No simple general theory describing the kinetics of solid-state chemical reactions exists. The diffusional transformations are controlled by a number of processes, such as interatomic diffusion, interface migration, the propagation of crystal defects, *etc*. However, often a simplified formula *x*(*t*) = 1 − exp(−*k* *t* *^n^*) is used to describe the growth of a new phase, where *x* is the fraction of the new phase, *t* is time, *n* and *k* are constants related to geometrical conditions of the grain boundaries and the rate of precipitation (Helgason *et al.*, 1999 ▸). We obtained the values of 316 (14) and 1.43 (1) for *k* and *n*, respectively (green dashed curve in Fig. 10 ▸).

Chemical kinetics in solid–gas systems has its own specifics. For example, it was observed that, due to the formation of a surface layer of reaction products on top of a solid, the kinetics typically has two regimes: a fast linear growth at the beginning and a slower parabolic region afterwards (Lee & Rapp, 1984 ▸). In the present study, the deviation from linear kinetics starts above ∼20% of conversion, which corresponds to approximately a 0.75 µm-thick layer of hematite on both sides of the iron foil. This value is quite close to 0.5 µm, reported as a typical layer thickness for intermetallics and silicides (Dybkov, 2002 ▸) when the reaction kinetics changes from linear to parabolic regimes.

## 6. Conclusion   

We present a new detector based on a CCD technology for fast time-resolved EXAFS spectroscopy at the ESRF with an acquisition rate in excess of 4 kHz. The performance of the new detector appears very attractive for a number of X-ray absorption spectroscopy applications, including *in situ* kinetics, shock wave experiments, pulsed field experiments and other time-resolved studies. As an example, a full time-resolved *in situ* XAS study of the oxidation of metallic iron in air at high temperature is presented. The existing implementation of the FReLoN system has an exposure time limited to 200 µs. Ongoing developments will allow exposure times to be reduced down to 30 µs, taking full benefit of the electronic shutter facility.

Pushing towards higher time resolution (shorter exposure times) would imply switching from a CCD technology to fast photodiodes (Headspith *et al.*, 2007 ▸). In principle, for even shorter exposures it is possible to use the time structure of a synchrotron storage ring. For example, if a single electron bunch is selected for measurements, time resolution of the order of 100 ps can be achieved even with a slow detector.

## Figures and Tables

**Table 1:** Performance of the different cameras Column 2 is for the old FReLoN camera based on a two-dimensional CCD chip, column 3 is for the new FReLoN camera equipped with the Hamamatsu S11165 array, and column 4 is for the Hamamatsu S11165 system produced by the original manufacturer.

Parameter	Old FReLoN camera	New FReLoN camera	Hamamatsu S11165
Pixel size (µm)	14 × 14	14 × 1000	14 × 1000
Full-well capacity (e^−^)	275000	297000	180000
Electronic noise (e^−^ r.m.s.)	19	23	45
Sensitivity (e^−^/ADU)	5	5.4	3
Dynamic range	1:15000	1:13000	1:4000
Integral nonlinearity	±0.4% of full range	±0.25% of full range	
Dark current (e^−^ pixel^−1^ ms^−1^)	0.001	2.3	100
Resolution (bit)	16	16	16
Full frame rate (frames s^−1^)	4	4230	1390
